# Use of Warning Signs for Dengue by Pediatric Health Care Staff in Brazil

**DOI:** 10.1371/journal.pone.0163946

**Published:** 2016-10-07

**Authors:** Luana Sicuro Correa, Yara Hahr Marques Hökerberg, Raquel de Vasconcellos Carvalhaes de Oliveira, Danielle Martins de Souza Barros, Helenara Abadia Ferreira Alexandria, Regina Paiva Daumas, Carlos Augusto Ferreira de Andrade, Sonia Regina Lambert Passos, Patrícia Brasil

**Affiliations:** 1 Department of Pediatrics, Rio de Janeiro State University, Rio de Janeiro, RJ, Brazil; 2 Laboratory of Clinical Epidemiology, Evandro Chagas National Institute of Infectious Diseases, Oswaldo Cruz Foundation, Rio de Janeiro, RJ, Brazil; 3 School of Medicine, Estácio de Sá University, Rio de Janeiro, RJ, Brazil; 4 Department of Obstetrics and Gynecology, Rio de Janeiro State University, Rio de Janeiro, RJ, Brazil; 5 Germano Sinval Faria Teaching Primary Care Center, National School of Public Health, Oswaldo Cruz Foundation, Rio de Janeiro, RJ, Brasil; 6 Acute Febrile Illnesses Laboratory, Evandro Chagas National Institute of Infectious Diseases, Oswaldo Cruz Foundation, Rio de Janeiro, RJ, Brazil; Institute of Tropical Medicine (NEKKEN), Nagasaki University, JAPAN

## Abstract

**Objective:**

The aim of this study was to describe the use of dengue warning signs by pediatric healthcare staff in the Brazilian public health care system.

**Methods:**

Cross-sectional study (2012) with physicians, nurses, and nurse technicians assisting children in five health care facilities. Participants reported the use and importance of dengue warning signs in pediatrics clinical practice through a structured questionnaire. Differences in the use of signs (chi-square test) and in the ranking assigned to each of them (Kruskal-Wallis) were assessed according to health care occupation and level of care (p<0.05).

**Results:**

The final sample comprised 474 participants (97%), mean age of 37 years (standard deviation = 10.3), mainly females (83.8%), physicians (40.1%) and from tertiary care (75.1%). The majority (91%) reported using warning signs for dengue in pediatrics clinical practice. The most widely used and highly valued signs were major hemorrhages (gastrointestinal, urinary), abdominal pain, and increase in hematocrit concurrent or not with rapid decrease in platelet count. Persistent vomiting as well as other signs of plasma leakage such as respiratory distress and lethargy/restlessness were not identified as having the same degree of importance, especially by nurse technicians and in primary or secondary care.

**Discussion:**

Although most health care staff reported using dengue warning signs, it would be useful to extend the training for identifying easily recognizable signs of plasma leakage that occur regardless of bleeding.

## Introduction

Dengue is currently a difficult-to-control global health problem, mainly affecting developing countries. The estimated burden of dengue in low and middle-income countries is 20 times that of developed countries [1]. In 2010, there were an estimated 96 million apparent infections, of which 14% occurred in the Americas, with half of these in Brazil [2].

The clinical presentation of dengue varies from a nonspecific febrile illness to severe forms with shock, representing different phases of the same disease [3,4]. Severe cases, although accounting for only 5% of all cases [4,5], constitute a high absolute number, presenting significant potential case fatality [2,6,7]. Thus, recognition and early intervention in potentially severe cases represent one of the principal strategies for reducing case fatality and increasing efficiency in the use of health resources [2,4,8].

Based on studies in various countries [9,10], the World Health Organization (WHO) proposed a new classification [4] aimed at improving the identification of severe forms and optimizing the clinical management of dengue. This classification emphasizes the recognition of warning signs as the principal risk marker of evolution to severity. Studies have shown that this new classification is easier to apply, since it can be used prospectively [11–13].

Although the proposed classifications apply to both adults and children, differences have been observed in dengue presentation between age groups [9]. Children represent a group of patients with peculiar characteristics, since dengue diagnosis and recognition of severe forms are both more difficult than in adults. Painful symptoms are generally poorly defined, especially in infants and preschoolers, and atypical symptoms such as coryza, cough, and diarrhea are similar to those found in other common childhood viral infections [8]. Studies suggest that evolution to severe forms tends to be more rapid in children, often without hemorrhagic manifestations or falling platelet count [14–17].

Few studies have described the knowledge and practices of health professionals involved in dengue care [18–20]. Previous studies in Taiwan, Singapore and Sri Lanka [19–21] have found gaps in health professionals’ knowledge on the clinical characteristics of dengue as well as a variability in clinical practice related to this disease. In Brazil, although the Ministry of Health has provided guidelines for diagnosis and treatment since 2007 [22], a study identified gaps in diagnosis and compliance with these guidelines in public health care services [18]. Thus, the aim of this study is to describe the use of warning signs for recognizing potential severe dengue in children self-reported by health professionals and technicians in the Unified National Health System (SUS).

## Methods

This was a cross-sectional and self-report study conducted from March to December 2012 in five health care services under the Unified National Health System in Rio de Janeiro: two primary care clinics with family health teams, one emergency service (secondary care), and two referral hospitals (tertiary care), both with specialized pediatric care.

The target population included physicians, nurses, and nurse technicians involved in care for children under 12 years with dengue (N = 544). The study excluded individuals without experience in assisting children with dengue, retirees, and those on work leave or vacation. The estimated minimum sample size was 464, considering 50% of prevalence, 95% confidence level, difference of 0.05 and allowing 20% of losses.

A pediatrician and two medical students were trained to contact the participants and deliver a self-applied questionnaire, which was returned in a sealed envelope after completion. The questionnaire is a Brazilian version [23] of the English questionnaire proposed by WHO after the study from Barniol et al. [11], which was adapted to Brazil according to the roadmap from Herdman et al. [24]. In the cross-cultural adaptation, the following warning signs were added as recommended by the guideline of the Brazilian Ministry of Health [8]: major hemorrhages (gastrointestinal, urinary), postural hypotension or syncope, decreased urine output, sudden drop in body temperature/hypothermia, respiratory distress, and painful hepatomegaly. Moreover, we followed the Brazilian dengue guideline, which considers increase in hematocrit concurrent or not to the rapid decrease in platelet count. The definitions of the dengue warning signs were in accordance with the Brazilian Ministry of Health [8] and WHO dengue guidelines [4].

The final version of the questionnaire contains the following information: sociodemographic (age and sex) and occupational (health care occupation and workplace) characteristics, experience in dengue care (yes/no), cumulative experience (<1 year, 1 to 5 years, >5 years), number of suspected dengue patients treated in the last year (none, 1 to 50, >50), and referral situation (“from primary care to the hospital”, “from the general hospital to the referral hospital”, “from the ward to the intensive care unit”). Participants in primary or secondary care were asked which criteria they used for referral in clinical practice (fever and rash, persistent vomiting, cold and clammy extremities, bleeding from nose or gums, thrombocytopenia ─ less than 100,000 platelet count, severe abdominal pain, any infant suspected of dengue, no predetermined criteria/ based on clinician’s judgment, other). All participants answered questions concerning overall use of warning signs (yes/no) and each specific warning sign (severe and continuous abdominal pain, abdominal tenderness, persistent vomiting, clinical fluid accumulation, mucosal bleeding, lethargy/restlessness, liver enlargement > 2 cm, laboratory: increase in hematocrit concurrent or not with rapid decrease in platelet count), in addition to the six signs adopted in Brazil) [8]. An item asked participants to rank a list of 12 warning signs according to the level of importance, ranging from 1 (more important) to 12 (less important). Finally, two open label/unstructured questions asked participants to add and/or remove signs from the list. We conducted a pilot study with the first 20 participants assigned to the study sample, who found the questionnaire easy to understand.

The data were keyed into EpiData. We described social and occupational variables as well as the use of warning signs according to level of care and occupation using Pearson’s chi-square test or Fisher’s exact test. We summarized the ranking assigned to each warning signs using quartiles and boxplot graphs. At first, we used Kruskal-Wallis non-parametric test to compare the ranking distribution across the occupational groups and level of care. After, we applied Mann-Whitney tests adjusted by Bonferroni method as a post-hoc test for ranking comparisons within pairs of those subgroups (e.g., physicians x nurses, physicians x nurse technicians and nurses x nurse technicians). Data analyses excluded missing data and were performed in the statistical package for social sciences—SPSS, version 16. Level of statistical significance for all tests was set at 5%.

### Ethics Statement

The study was approved by the research ethics committee of the National Institute of Infectious Diseases Evandro Chagas of the Oswaldo Cruz Foundation (CAAE: 0032.0.009.000–11), the Pedro Ernesto University Hospital, State University of Rio de Janeiro (3100/2011-CAAE: 0238.0.228.009–11), the Municipal Department of Health and Civil Defense of Rio de Janeiro (CAAE: 0035.0.009.314–11) and the National Institute of Women, Children and Adolescents Health Fernandes Figueiras of the Oswaldo Cruz Foundation (FR448158). All participants signed the free and informed consent form.

## Results

**Fig 1:**
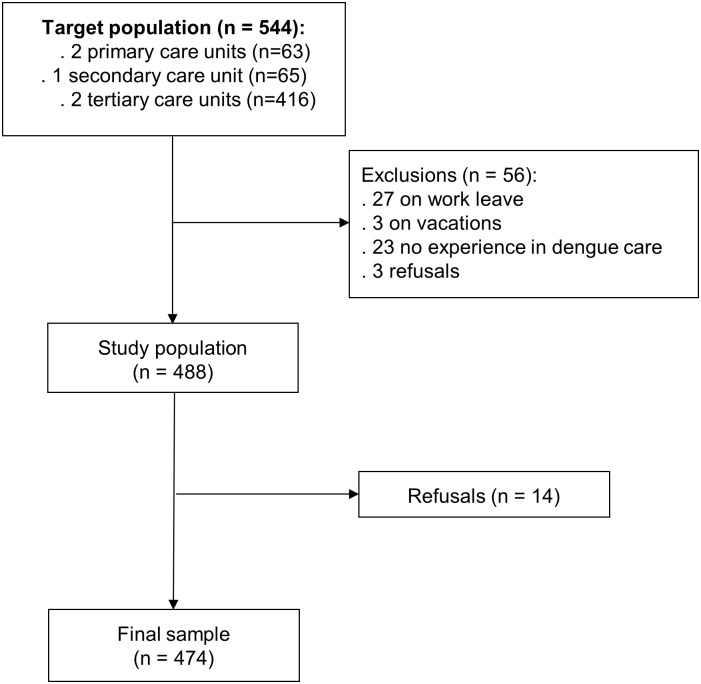
Flowchart of sample selection.

Of the total of 544 participants, 488 met the inclusion criteria, and 474 (97%) answered the questionnaire (Fig 1). Mean age was 37 years (standard deviation: 10.3), the majority were females (83.8%), physicians (40.1%) and from the tertiary care (75.1%) (Table 1).

**Table 1:** Sociodemographic characteristics and experience in dengue care (n = 474).

Variables—n(%)	n (%)
Females	397 (83.8)
Occupation	
Physicians	190 (40.1)
Nurses	108 (22.8)
Nurse technicians	176 (37.1)
Level of care	
Primary (Family health teams)	58 (12.2)
Secondary (Emergency service)	60 (12.7)
Tertiary (Referral hospitals)	356 (75.1)
Cumulative experience in dengue care ^1^	
< 1 year	94 (19.9)
1 to 5 years	251 (53.3)
> 5 years	126 (26.8)
Suspected dengue patients in the last year ^2^	
None	26 (5.5)
1 to 50	371 (78.6)
> 50	75 (15.9)

^1^ 3 missing values,

^2^ 2 missing values

At the primary and secondary care levels, “bleeding from the nose or gums” (respectively 84% and 90%, p = 0.31) and “severe and continuous abdominal pain” (respectively 88% and 87%, p = 0.59) were the signs more frequently used to refer patients to higher level of care. “Fever and rash” was the least frequently used sign. Presence of “cold and clammy extremities” was the criterion for referral adopted by 58% of participants at the primary care level and 55% at the secondary level (p = 0.18). When compared to primary care services, health professionals at the secondary level used the following criteria more frequently (p < 0.05) for referring patients to higher levels of care: “infant with suspected dengue” (primary care: 43% and secondary care: 63%, p = 0.04), “fever and rash” (respectively, 21% and 42%, p = 0.03) and “no predetermined criterion, referral based on clinical assessment” (respectively, 12% and 22%, p = 0.02).

In the total sample, 90.7% reported using the warning signs recommended by WHO (2009) and the Brazilian Ministry of Health (2011), with no statistically significant difference between levels of health care (p = 0.66). At all three levels of care, the most frequently used sign was “major hemorrhages”, followed by “severe and continuous abdominal pain” and “increase in hematocrit concurrent or not with rapid decrease in platelet count” (p > 0.05). The signs “decreased urine output”, “persistent vomiting”, and “abdominal tenderness” were more frequently used at the tertiary level, while “bleeding from nose or gums” at the secondary level (Table 2).

**Table 2:** Use of dengue warning signs in children according to level of health care (n = 474).

Warning signs	Primary care	Secondary care	Tertiary care	P value^1^
	N = 58	N = 60	N = 356	
	n (%)	n (%)	n (%)	
Overall use of warning signs	51 (91)^2^	57 (95)	323 (92)^3^	0.660
Major hemorrhages	55 (98)^2^	58 (97)	332 (94)^4^	0.263
Severe and continuous abdominal pain	54 (93)	53 (88)	331 (93)	0.444
Rising hematocrit and/or falling platelet count	53 (91)	51 (85)	323 (91)	0.366
Bleeding from nose or gums	47 (81)	55 (92)	260 (73)^2^	**0.006**
Hypotension /syncope	46 (81)^4^	46 (77)	305 (86)	0.166
Lethargy /restlessness	44 (77)^4^	45 (75)	290 (82)^2^	0.367
Rapid decrease in body temperature	44 (77)^4^	46 (77)	235 (66)^4^	0.094
Decreased urine output	43 (77)^2^	36 (60)	294 (83)^4^	**<0.001**
Respiratory distress	44 (76)	41 (68)	279 (78)	0.231
Body cavity effusions^5^	43 (75)^4^	38 (64)^4^	249 (70)^2^	0.428
Persistent vomiting	40 (69)	39 (65)	277 (78)^2^	**0.042**
Painful liver enlargement	36 (64)^2^	29 (48)	210 (60)^6^	0.164
Abdominal tenderness	23 (40)^4^	18 (30)	197 (56)^7^	**<0.001**

^1^P-value of Chi-squared test;

^2^ 2 missing values;

^3^ 4 missing values;

^4^ 1 missing value;

^5^ ascites, pleural and pericardial effusions;

^6^ 6 missing values;

^7^ 3 missing values.

Table 3 shows that most physicians reported using “major hemorrhages”, “lethargy/restlessness” and “body cavity effusions”. Nurses more frequently used “severe and continuous abdominal pain”, major hemorrhages and rising hematocrit/falling platelet count”, while nurse technicians used mostly “bleeding from nose or gums”, “rising hematocrit/falling platelet count” and “major hemorrhages”. Most of the warning signs showed statistically significant differences in their use according to health care occupation. Except “rising hematocrit and/or falling platelet count”, “bleeding from nose or gums” and “abdominal tenderness”, all other warning signs were significantly more frequently used by physicians than by nurses and nurse technicians (p<0.001).

**Table 3:** Use of dengue warning signs in children by health care occupations (n = 474).

Warning signs^1^	Physicians	Nurses	Nurse technicians	Total	P value^2^
	(N = 190)	(N = 108)	(N = 176)	(N = 474)	
	N(%)	N(%)	N(%)	N(%)	
Major hemorrhages	188 (99)	99 (92)	158 (91) ^3^	445 (94) ^3^	**0.002**
Severe and continuous abdominal pain	180 (95)	100 (93)	158 (90)	438 (92)	0.200
Rising hematocrit and/or falling platelet count	168 (88)	95 (88)	164 (93)	427 (90)	0.221
Hypotension /syncope	180 (95)	87 (81)	130 (74) ^4^	397 (84) ^4^	**<0.001**
Lethargy /restlessness	187 (98)	81 (75)	111 (64) ^3^	379 (80) ^3^	**<0.001**
Decreased urine output	168 (88)	82 (77) ^4^	123 (71) ^5^	373 (79) ^3^	**<0.001**
Respiratory distress	171 (90)	82 (76)	111 (63)	364 (77)	**<0.001**
Bleeding from nose or gums	118 (62)	81 (75)	163 (94) ^5^	362 (77) ^5^	**<0.001**
Persistent vomiting	151 (80)	77 (71)	128 (74) ^5^	356 (75) ^5^	0.223
Body cavity effusions ^6^	183 (96)	68 (63)	79 (46) ^7^	330 (70) ^7^	**<0.001**
Rapid decrease in body temperature or hypothermia	159 (84) ^4^	64 (60) ^4^	102 (58)	325 (69) ^5^	**<0.001**
Painful liver enlargement	132 (70) ^4^	61 (58) ^3^	82 (48) ^7^	275 (59) ^8^	**<0.001**
Abdominal tenderness	70 (37) ^4^	51 (48) ^5^	117 (67) ^4^	238 (51) ^7^	**<0.001**

^1^ List of clinical and laboratorial data defined by WHO (2009);

^2^ P-value of the Chi-squared test;

^3^ 3 missing values;

^4^ 1 missing value;

^5^ 2 missing values;

^6^ ascites, pleural and pericardial effusions;

^7^ 4 missing values;

^8^ 8 missing values.

**Fig 2:**
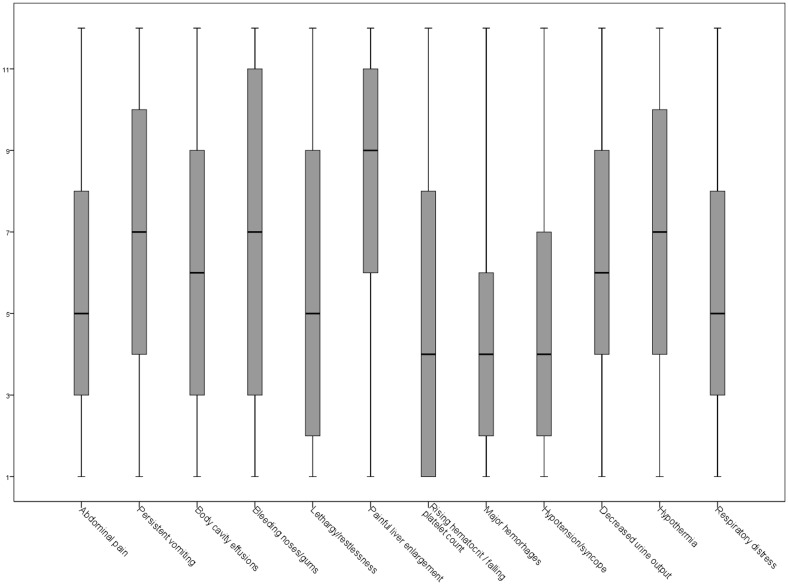
Ranking assigned to dengue warning signs in children.

When ranked, the signs with median punctuation lower than 4, that is, the most relevant, were “major hemorrhages”, “increase in hematocrit concurrent or not with rapid decrease in platelet count”, and 'postural hypotension and/or syncope”. However, a wide range was observed in the ranking assigned to each sign, while “major hemorrhages” and “painful liver enlargement” showed the smallest variation in punctuations (Fig 2).

The assigned rankings showed differences between health care occupations (Table 4). Physicians tended to value “hypotension and/or syncope” and “lethargy/restlessness” when compared to other health workers (p<0.01). Nurse technicians tended to value “rising hematocrit and/or falling platelet count” and “bleeding from the nose or gums” more than physicians and nurses (p<0.01).

**Table 4:** Ranking^1^ assigned to each dengue warning sign in children according to health care occupations (n = 471)^2^.

Warning signs	Physicians	Nurses	Nurse technicians	P-value^3^
	Median (IQI)	Median (IQI)	Median (IQI)	
Hypotension /syncope ^4^^,^^5^	2 (1–5)	6 (3–8)	6 (3–8)	<0.001
Lethargy /restlessness ^4^^,^^5^	2.5 (1.8–5)	7 (4–9)	7 (4–10)	<0.001
Major hemorrhages (gastrointestinal, urinary)^5^	4 (2–6.2)	3 (2–6)	3 (2–5)	0.021
Respiratory distress^5^	5 (3–7.2)	5 (2–9)	6.5 (3–9)	0.049
Decreased urine output ^4^^,^^5^	5 (3–8)	7 (5–9)	8 (5–10)	<0.001
Body cavity effusions	5.5 (3–8)	5 (2–10)	6 (3–9)	0.156
Rapid decrease in body temperature/hypothermia^5^	6 (4–9)	8 (5–10.8)	8 (5–11)	0.002
Rising hematocrit and/or falling platelet count^4^^,^^5^^,^^6^	7 (3–10)	4 (1–8)	2 (1–4)	<0.001
Severe/continuous abdominal pain^4^^,^^5^	7 (4–9)	4,5 (2–7.8)	4 (2–7)	<0.001
Persistent vomiting^5^^,^^6^	8 (5–10)	7 (4–10)	5 (3–8.8)	<0.001
Bleeding from nose/gums^4^^,^^5^^,^^6^	10 (6.8–12)	7 (4–10.8)	3 (2–6)	<0.001
Painful liver enlargement^4^^,^^5^^,^^6^	10 (8–11)	9 (6–11)	8 (5–10)	<0.001

IQI—Interquartile interval.

^1^ Ranking ranges from 1 (more important) to 12 (less important);

^2^ 3 missing values;

^3^Kruskal-Wallis test;

^4^ p < 0.05 of Mann-Whitney test (physicians x nurses);

^5^ p < 0,05 of Mann-Whitney test (physicians x nurse technicians);

^6^ p < 0,05 of Mann-Whitney test (nurses x nurse technicians).

Ninety-seven participants suggested adding other warning signs to the list, such as fever, prostration, myalgia, headache, arthralgia, rash, retro-orbital pain, and positive tourniquet test. Forty-eight participants would have added petechiae, while 44 would have added the following signs of shock: narrow pulse pressure, fine pulse, alteration in pulse amplitude, sluggish capillary filling, and tachycardia. Meanwhile, some participants suggested removing from the list painful hepatomegaly (n = 66), bleeding from nose or gums (n = 44), rapid decrease in temperature (n = 33), pleural effusion and ascites (n = 33), respiratory distress (n = 21), decreased urine output (n = 15), and abdominal pain (n = 16).

## Discussion

In the current study, the majority of the health professionals and technicians caring for children reported using warning signs for severe dengue in clinical practice. Bleeding, severe and continuous abdominal pain and rising hematocrit concurrent or not with falling platelet count were the most highly valued signs, especially by nurse technicians and in services with lower complexity of health care. Comparatively, other warning signs such as respiratory distress and persistent vomiting were not reported with the same degree of importance.

Health professionals and technicians working in primary (88%) and secondary care (78%) reported using some predetermined criteria for referring patients. This is probably due to the fact that periodic training courses were based on the Brazilian guideline that recommends the use of warning signs [8] similar to those listed on the WHO guidelines [4]. In contrast, in a study in Sri Lanka [21], only 45% of clinicians and 40% of pediatricians reported using WHO guidelines.

The majority of our participants identified “major hemorrhage” as a relevant warning sign in pediatrics clinical practice, regardless of their health care occupation or level of care, in agreement with other studies [25,26]. However, heterogeneity in the definitions of bleeding and hemorrhage highlight the low specificity of these signs as indicators of the need for intervention, as demonstrated in the literature [17,27–31].

“Bleeding from the nose and gums” were more valued in secondary care, while “decreased urine output” was the most frequently used sign in tertiary care. This suggests that health professionals in emergency care associated severity more with bleeding than with indirect signs of hypovolemia.

The fact that severe and continuous abdominal pain is a frequently used sign in our sample is consistent with studies that showed its association with plasma leakage, indicating potential evolution to severity [5,31–35]. However, at least 20% of respondents failed to value other signs of plasma leakage such as body cavity effusions, respiratory distress, “decreased urine output” and lethargy/restlessness, as well as persistent vomiting and painful hepatomegaly. Only 46% of nurse technicians and 63% of nurses reported the use of “body cavity effusions” since its identification is beyond their professional duties and competences. Respiratory distress, easy for any health professional to detect, can indicate both the presence of pleural effusion and hemodynamic instability. Studies have reported an association between presence of lethargy, severe abdominal pain, and body cavity effusions at admission in patients with dengue that evolved to severity, not always accompanied by hemorrhage [16,17,25,26,29,33,35–37]. Lethargy is not only a sign of hypovolemic shock, but can also indicate encephalopathy, an atypical and severe presentation of dengue in children [38–40].

Increase in hematocrit concurrent or not with decrease in platelet count was the third most frequently identified sign in our study. This is probably due to the fact that these signs have been included in the definition of dengue hemorrhagic fever since 1997 [3]. The perception of low platelet count as a sign of severity in dengue is consistent with a study on primary care physicians’ knowledge in Singapore, where 52% reported using platelet count below 80,000 as a criterion for hospitalization [20].

Analysis of signs ranking shows the importance assigned to hemorrhagic manifestations as warning signs in children. The lack of recognition of other signs of plasma leakage in the absence of bleeding, mainly by nurses and nurse technicians is a reason for concern. It is also known that children’s evolution to shock can be fast and without hemorrhagic manifestations [14–17]. In contrast, physicians considered more important signs of severity related to the pathophysiology of the critical phase of dengue as postural hypotension/syncope and lethargy/restlessness, showing that this occupational group has more familiarity with the severe forms of the disease. As the revised WHO classification (2009) hallmarks, the understanding of dengue's pathophysiology has also changed; it is now recognized as plasma leakage-related rather than hemorrhage-related.

In Brazil, nurses and nurse technicians play a broader role in health care [41–43], especially in primary care and in emergency care units, where they are responsible for triage. Nurse technicians represent the largest contingent of health workers [42] and are usually in direct contact with patients during admission. They also play an important role in the community, as members of family health teams as well as in underserved areas where they usually are the only social agents with relevant technical information for clinical management of dengue cases. Nonetheless, in the five health care facilities analyzed in our study, the nurse technicians were not included in training for dengue management.

The wide variation observed in rankings assigned to warning signs may be explained by the fact that health professionals used to evaluate syndromes rather than the single role of each sign or symptom.

Knowledge gaps among health professionals concerning the clinical characteristics of dengue and variation in approaches have also been found in studies in Taiwan, Singapore, and Siri Lanka [19–21]. To our knowledge, this is the first study outside Asia that evaluated the routine use of warning signs and symptoms for dengue in children by health professionals and technicians. Our results underscore the importance of training health staff in the revised dengue classification proposed by the WHO (2009), in which warning signs are the most important aspect for identifying potentially severe cases.

The fact that 75% of the sample included workers from referral hospitals limits the generalization of our findings. However, both hospitals included in this study have outpatient and emergency care services. Since Brazilian health care workers often hold multiple jobs, it is reasonable to assume that many of the interviewees also work in health care facilities at different levels of complexity.

Based on this study, we can infer that major hemorrhages, rising hematocrit, falling platelet count, and severe and continuous abdominal pain are the most widely used warning signs for severe dengue in children, especially in primary care and by nurses and nurse technicians. Training that extends to all health professionals and technicians involved in triage and clinical management of dengue would be useful for identifying easily recognizable signs of plasma leakage, such as respiratory distress, persistent vomiting, and lethargy/restlessness. Future studies should be conducted in different settings to confirm our findings. Our results can contribute to new approaches for training physicians, nurses and nurse technicians, considering their specific roles in health care, as well as to the assessment and review of management strategies, which are necessary for optimizing care.
